# Supplementary material for: Angelica sinensis polysaccharide as potential protectants against recurrent spontaneous abortion: focus on autophagy regulation
Source: Front Med (Lausanne). 2025 Jan 15;12:1522503. doi: 10.3389/fmed.2025.1522503 (PMC11774876; doi:10.3389/fmed.2025.1522503)
Supplement: Supplementary material S1 — The main instruments used during the LC-MS process, along with their models/specifications and manufacturers. [file Supplementary_file_1.zip › Metabolomics sequencing data FC1.2/8.代谢通路分析/Exposure_Ctrl/all/KEGG/mmu00440.html]

KEGG PATHWAY: Phosphonate and phosphinate metabolism - Mus musculus (house mouse)


# Phosphonate and phosphinate metabolism - Mus musculus (house mouse)


[
Pathway menu
| Organism menu
| Pathway entry
| Show description
| Download
| Help
]

Natural products containing carbon-phosphorous bonds, so-called C-P compounds, are derivatives of phosphonate and phosphinate with substitution of alkyl group for hydrogen of phosphorus-hydrogen bonds. C-P compounds have been found in many organisms, but only protists and bacteria, mostly Actinobacteria, have biosynthetic capacity. A common reaction in the biosynthetic pathway is C-P bond forming reaction from phosphoenolpyruvate (PEP) to phosphonopyruvate (PnPy) catalyzed by PEP phosphomutase. 2-Aminoethylphosphonate (AEP) is the most abundant C-P compound in the natural world. AEP derivatives include phosphonoprotein, phosphonoglycan, and phosphonolipid. Other known C-P compounds are bioactive substances used in medicine (antibiotics) and agriculture (herbicide) such as fosfomycin, FR-33289, rhizocticin, and bialaphos.


##### Option

Scale:


100%

Image resolution:


 High

##### Search

##### ID search

##### Color


KGML

Image (png) file 1x
